# Reducing bottlenecks: professionals’ and adolescents’ experiences with transitional care delivery

**DOI:** 10.1186/1472-6963-14-47

**Published:** 2014-01-31

**Authors:** Anna P Nieboer, Jane M Cramm, Henk M Sonneveld, Marij E Roebroeck, AnneLoes van Staa, Mathilde MH Strating

**Affiliations:** 1 Institute of Health Policy & Management (iBMG), Erasmus University, Rotterdam, The Netherlands; 2 Department of Rehabilitation Medicine and Physical Therapy, Erasmus University Medical Centre, Rotterdam, The Netherlands; 3 Centre of Expertise Innovations in Care, Rotterdam University, Rotterdam, The Netherlands

**Keywords:** Bottlenecks, Quality of care, Transitional care, Transition programme, Quality improvement programme, Adolescents

## Abstract

**Background:**

The purpose of this study was to describe the interventions implemented in a quality improvement programme to improve transitional care and evaluate its effectiveness in reducing bottlenecks as perceived by professionals and improving chronically ill adolescents’ experiences with care delivery.

**Methods:**

This longitudinal study was undertaken with adolescents and professionals who participated in the Dutch ‘On Your Own Feet Ahead!’ quality improvement programme. This programme followed the Breakthrough Series improvement and implementation strategy.

A total of 102/128 (79.7%) professionals from 21 hospital teams filled out a questionnaire at the start of the programme (T0), and 79/123 (64.2%; five respondents had changed jobs) professionals completed the same questionnaire 1 year later (T1). Seventy-two (58.5%) professionals from 21 teams returned questionnaires at both time points. Of 389 and 430 participating adolescents, 36% and 41% returned questionnaires at T0 and T1, respectively. We used descriptive statistics and two-tailed, paired t-tests to investigate improvements in bottlenecks in transitional care (perceived by professionals) and care delivery (perceived by adolescents).

**Results:**

Professionals observed improvement in all bottlenecks at T1 (vs. T0; p < 0.05), especially in the organisation of care, such as the presence of a joint mission between paediatric and adult care, coordination of care, and availability of more resources for joint care services. Within a 1-year period, the transition programme improved some aspects of patients’ experiences with care delivery, such as the provision of opportunities for adolescents to visit the clinic alone (p < 0.001) and to decide who should be present during consultations (p < 0.05).

**Conclusions:**

This study demonstrated that transitional care interventions may improve the organisation and coordination of transitional care and better prepare adolescents for the transition to adult care within a 1-year period. By setting specific goals based on experiences with bottlenecks, the breakthrough approach helped to improve transitional care delivery for adolescents with chronic conditions.

## Background

The importance of improving chronically ill adolescents’ experiences with transitional care has been increasingly acknowledged [1-13]. Blum et al. [4] defined transitional care as the purposeful, planned movement of adolescents and young adults with chronic physical and medical conditions from child-centred to adult-oriented health care systems. This multifaceted, active process must attend to the medical, psychosocial, educational, and vocational needs of chronically ill adolescents. While various types of transition programmes have been proposed [14], there is no evidence that particular models of transition are more effective than others [2,15]. Consequently, there is significant variability in transition support provided to adolescents [16]. Programme expectations are largely agreed upon [17], but we lack empirical evidence related to transitional care design and effectiveness in improving experiences with care delivery [2]. Although most transition programmes consist of a constellation of interventions, most studies have examined these interventions in isolation, without considering the multifaceted nature of the programmes and the concurrent impacts of multiple interventions. Furthermore, the specific details of programme content are rarely described and very few studies have applied and evaluated transition interventions in practice [15], highlighting the need for further research to determine the impact of these programmes on adolescents’ and professionals’ experiences with care delivery [7].

In the Netherlands, transitional care is not structured and usually takes place on an ad hoc basis. Furthermore, van Staa and colleagues [18,19] found that essential elements of transitional care, such as transition protocols, coordinators, and individual transition plans, are largely lacking and that improvement of transitional care was regarded as highly necessary both by professionals, parents and adolescents themselves. The Dutch quality improvement programme ‘Op Eigen Benen Vooruit!’ (On Your Own Feet Ahead!) was designed to reduce bottlenecks and improve transitional care through the development and implementation of appropriate interventions for adolescents with chronic physical conditions in hospitals and rehabilitation centres. The purpose of this study was to describe the interventions implemented in the quality improvement programme and evaluate its effectiveness in reducing bottlenecks as perceived by professionals and improving chronically ill adolescents’ experiences with care delivery.

## Methods

### ‘On your Own feet ahead!’ quality improvement programme

This longitudinal study was undertaken with adolescents and professionals participating in the ‘On Your Own Feet Ahead!’ quality improvement programme to examine the implementation of transitional care interventions in rehabilitation centres and hospitals for adolescents with chronic conditions in the Netherlands. This programme followed the Breakthrough Series improvement and implementation strategy [20]. We previously reported adolescents’ , parents’ , and providers’ perceptions of transitional care at the start of the programme; all participants reported that there was room for improvement [21]. Adolescents and parents indicated that the care process offers most room for improvement. Providers also reported shortcomings in the care delivery process, especially with respect to guidelines, protocols and coordination. In addition, providers reported that the adolescents’ responsibility in care is (very) often lacking and that parents have trouble ceding control to their children. Furthermore, we investigated relationships between general self-efficacy (perceived by adolescents with chronic conditions and their parents) and adolescents’ quality of life [22,23], and between satisfaction with care and self-efficacy and quality of life among adolescents with diabetes [24]. These studies highlighted the importance of self-efficacy and satisfaction with care in the quality of life of chronically ill adolescents. However, we have not yet reported on transition programme design or effectiveness in improving experiences with care delivery, as perceived by adolescents and professionals. The current study adds to this knowledge by providing detailed descriptions of interventions implemented in the quality improvement programme and evaluating its effectiveness over time in reducing bottlenecks and improving chronically ill adolescents’ experiences with care delivery, which has not been done before.

### Transitional care interventions

At the start of the ‘On Your Own Feet Ahead!’ programme, professionals involved in care delivery to chronically ill patients identified bottlenecks in transitional care provided by their multidisciplinary team, and then designed and agreed on a specific plan of action. For this, a comprehensive model for transitional care delivery was used [25]. In the course of the 1-year programme, the teams implemented local interventions to improve transitional care in the identified areas of concern. Within the context of the quality improvement collaborative consultants and experts (including medical professionals) offered guidance through on-site visits, telephone and email conversations from the first step [setting (clinical) goals] to the final step (evaluating the effects of efforts to improve care) of the programme. In addition, professionals involved in care delivery to chronically ill patients were invited to participate in a total of seven learning sessions in which team building, collaboration, and dealing with barriers in the local setting were emphasised.

During the course of the programme, a toolkit with descriptions of approximately 30 suitable and effective interventions for adolescents with chronic conditions was developed and made available on the project’s website (http://www.opeigenbenen.nu). Professionals were supplied with formats, instruments, and examples of each intervention.

Three rounds (one pilot round and two dissemination rounds) of 10 teams each participated in the quality improvement programme (November 2009 – May 2012). In the first round, one rehabilitation team left the programme due to lack of management support and one hospital team was delayed due to changes in personnel. The remaining 21 hospital teams and seven rehabilitation centre teams implemented several transitional care interventions targeted at diverse patient populations: adolescents with type I diabetes, juvenile idiopathic arthritis (JIA), cystic fibrosis, chronic kidney failure and/or kidney transplantation, congenital urological conditions, and neuromuscular disorders (NMDs); the latter used home ventilation. Rehabilitation centre teams treated childhood disabilities, including cerebral palsy, spina bifida, and NMDs.

Each team used a combination of several interventions to improve the organisation of transitional care and to enhance the self-management of adolescents with a chronic condition.

These included (1) designing a protocol and joint mission for the transition from paediatric to adult care, (2) appointing a transition coordinator, (3) improving multidisciplinary consultation between paediatric and adult care and/or implementing a transition (outpatient) clinic, (4) implementing an individual transition plan (ITP), and (5) challenging adolescents to attend consultations independently (without parents present). Appendix 1 provides a detailed overview of interventions implemented by each team.

### Study design and procedure

The current study describes the efforts of all hospital-based teams (n = 21) that followed a sequential transition model [2] in which services were constructed as an extension of paediatric care provision or provided jointly by adult and paediatric care professionals. We do not examine the efforts of rehabilitation centre–based teams here because they mainly followed a developmental transition model with an active focus on adolescents’ personal growth and development, aiming to integrate social, educational, and assisted living services provided in these centres. This differs from transitional care services in hospitals.

Project leaders from 21 teams (each team represents a different organization) selected 4 to a maximum of 10 team members (depending on the type of setting) to fill out the questionnaire used in this study. Teams tried to involve professionals from paediatric as well as adult care. Each selected team member received a questionnaire by mail at the beginning of the programme (T0) and one year later (T1).

At T0, adolescents with chronic conditions were asked in writing to complete a questionnaire. Informed consent for participation in the study was obtained from parents as well as adolescents. A reminder was sent to non-respondents 2 weeks later. Inclusion criteria for the questionnaire-based survey of adolescents were: age 11–25 years, no history of a mental disorder, and receipt of paediatric care or treatment at T0. At T1, all adolescents were asked to complete the questionnaire again. They were offered a small financial incentive for participation (the adolescents from the test phase groups were entered in a lottery with a chance to win an iPod, and the other groups received two tickets to the cinema or a gift certificate).

### Ethics

This study was approved by the Medical Ethics Committee of Erasmus University Medical Centre, Rotterdam.

### Measures

#### Professionals

In addition to soliciting demographic data, the questionnaire administered to professionals asked them to rate the presence/frequency of bottlenecks (shortcomings) in transitional care. These items were selected on the basis of a quick review of relevant literature [19,26,27] because no existing instrument measured providers’ experiences with care delivery. Respondents were asked to rate 35 items representing eight types of bottlenecks (four or five items per subscale) using a five-point Likert scale ranging from 1 (never) to 5 (very often). Examples of items are ‘parents have trouble ceding control to adolescents’ , ‘there is a lack of coordination between paediatric and adult care’ , and ‘adolescents take too little responsibility for self-care’. Total scores were calculated by summing responses to individual items, with higher scores indicating greater shortcoming. Cronbach’s alpha coefficients for these subscales ranged from 0.69 to 0.88 in this study.

#### Adolescents

In addition to background characteristics, the questionnaire administered to adolescents included the ‘Mind the GAP’ scale, which measures quality aspects of current care delivery [28]. The instrument consists of 22 items, with responses structured by a seven-point Likert scale ranging from 1 (strongly disagree) to 7 (strongly agree). In the current study, we used 14 of these items, which were closely linked to expected changes in the quality of care delivery as a result of the quality improvement programme.

### Statistical analyses

All statistical analyses were conducted with SPSS software (ver. 18.0; IBM). We used descriptive statistics and two-tailed, paired t-tests to investigate improvements in bottlenecks in care (perceived by professionals) and care delivery (perceived by adolescents). Only data from participants who completed the questionnaire at both time points was analysed.

## Results

### Characteristics of professionals

A total of 102/128 (79.7%) professionals representing the 21 hospital-based teams completed the questionnaire at T0, and 79/123 (64.2%; five respondents had changed jobs at T1) professionals representing 21 teams completed the questionnaire at T1. Seventy-two (58.5%) professionals representing 21 teams returned questionnaires at both time points. Participating professionals included 17 medical specialists (including paediatricians) working in paediatric care, 7 medical specialists working in adult care, 28 specialized nurses, 7 nurse practitioners, 7 social workers, 2 psychologists, 1 developmental psychologist, 2 dieticians and 1 quality manager.

### Characteristics of adolescents

Of 389 (T0) and 430 (T1) participating adolescents, 36% completed the questionnaire at T0 and 41% at T1. About half (53%) of the respondents were female and their mean age was 16.1 ± 2.3 years at T0 (range 11–25) and 17.4 ± 2.3 years at T1 (range 12–26). About 18% of respondents reported having JIA, 62% diabetes, 5% cystic fibrosis or another lung disease, 7% a kidney condition, 4% a urological condition, and about 5% a NMD.

### Bottlenecks in transitional care

Table 1 provides an overview of bottlenecks in transitional care perceived by professionals. At T0, professionals were concerned about the lack of a joint mission for and coordination between paediatric and adult care. Parents’ difficulties with ceding control to adolescents and adolescents’ insufficient responsibility for self-care were also reported as bottlenecks. Providers also mentioned adolescents’ psychosocial problems, non-compliance, and social participation. At T1, professionals perceived significant (p < 0.05) improvement in all bottlenecks. The most pronounced improvements were perceived in the organisation of care, such as the existence of a joint mission between paediatric and adult care, coordination of care, and the availability of more resources for joint care services.

**Table 1:** Bottlenecks in transitional care perceived by professionals (n = 72)

	**Mean (SD) at T0**	**Mean (SD) at T1**	**Change (T0 – T1) (SD)**	***p***
No joint mission between paediatric and adult care	3.4 (1.0)	1.8 (0.6)	1.6 (0.9)	<0.001
Parents have trouble ceding control to adolescents	3.1 (0.7)	2.6 (0.6)	0.5 (0.7)	<0.001
Lack of coordination between paediatric and adult care	3.0 (0.8)	1.8 (0.4)	1.2 (0.8)	<0.001
Adolescents take too little responsibility for self-care	2.9 (0.7)	2.5 (0.5)	0.4 (0.6)	<0.001
Lack of resources for joint care services	2.7 (0.9)	1.8 (0.6)	0.9 (1.0)	<0.001
Psychosocial problems of adolescents	2.7 (0.5)	2.5 (0.5)	0.2 (0.5)	0.027
Non-compliance of adolescents with therapy	2.7 (0.7)	2.4 (0.5)	0.3 (0.6)	<0.001
Social participation of adolescents	2.3 (0.6)	2.2 (0.6)	0.2 (0.5)	0.005

SD, standard deviation; T0, beginning of ‘On Your Own Feet Ahead!’ quality improvement programme; T1, one year later. Only data from respondents who completed the questionnaire at both T0 and T1 were included in the analyses.

### Transitional care interventions

An additional file describes the interventions implemented by each team [see Additional file 1].

As examples, detailed descriptions of the efforts of the nephrology team at St. Radboud University Medical Centre (UMC) and the diabetes team at the VU University Medical Center Amsterdam are provided below [25].

### The nephrology team at UMC St. Radboud (Radboud University Nijmegen Medical Centre)

In 2010, the nephrology team at UMC St. Radboud, Nijmegen, joined the quality improvement collaborative. Their main goal was to improve transitional care for adolescent kidney transplant recipients who require life-long medical support. About 5–10 such adolescents transition from paediatric to adult care each year at UMC St. Radboud. A nurse practitioner was appointed as transition coordinator. She oversees the transition process and maintains close contact with the adolescents. ‘I consider it my responsibility to inform the other team members, parents, and adolescents, and to make sure that the interventions planned at the outpatient clinic are actually carried out. Together with the other team members, we think of ways to list the interventions in the electronic patient file. It is my responsibility to track those children who are in the transition from paediatric to adult care and to set up appointments for them to join the transition programme. Currently, we work with an Individual Transition Plan for the 12- to 16-year-olds. For the 16- to 18-year-olds, we work with ITPs as well as group meetings.’

Adolescents from throughout the Netherlands visit the nephrology department. Some adolescents transition to a different hospital located in their region, which makes joint transition policies more difficult. This problem is addressed in part by asking adolescents to return to Nijmegen for at least a year during the transition period. All adolescents attended the first group meeting, regardless of travel distance, reflecting their involvement in the transition process. ‘There is strong transition awareness in the entire team. Adolescents, parents, and us professionals, we are all growing in this process’.

### The diabetes team at VU University Medical Center, Amsterdam

The diabetes team at VU University Medical Center in Amsterdam treats about 50 adolescents aged 12–19 years with type I diabetes mellitus. In the first half year of the transition programme, professionals prepared a series of interventions to improve the transition process; these interventions were implemented thereafter. ITPs were developed for all adolescents aged > 12 years, a transition outpatient clinic was set up in collaboration with the internal medicine team, and group consultations on relevant themes were organised. Team members followed a motivational interviewing course. The project leader, a specialised paediatric diabetes nurse, explained ‘Before the transition programme there was no transitional care. We told the children, “you are 18 years old now and you have to cross over”’.

The team began using a questionnaire developed specifically for adolescents with type 1 diabetes to monitor their physical, emotional, social, and intellectual quality of life in a structured way. Adult care (internal medicine) professionals did not participate initially in the project, but they also eventually joined the team. The team was able to improve transitional care within the specified time frame. The pressure created by defining this time frame actually helped professionals to realise the ambitious goals of the transition programme.

To enhance adolescents’ self-management, most teams asked them to complete ITP forms, thereby setting goals in several domains. They provided adolescents with written or online information about transitional care. Professionals used checklists to monitor adolescents’ transitions, for example by recording their developmental stages in different domains. They also arranged to see adolescents aged > 16 years independently (without parents) for most of the time during consultations.

Interventions to improve the organisation of transitional care mainly involved the creation of transition protocols and written plans and schedules of professionals’ tasks and responsibilities. Some teams appointed transition coordinators, paediatric or adult care professionals dedicated to monitoring adolescents’ transitions. In most cases, paediatric and adult care professionals developed joint policies and missions to align procedures and treatment protocols. Several teams were also able to organise transition clinics in which paediatric and adult care professionals saw patients jointly. Structured consultations between paediatric and adult care professionals with regular multidisciplinary discussions about transitioning patients were also common. Finally, some teams implemented group consultations, such as shared medical appointments or peer group education sessions.

### Adolescents’ experiences with transitional care

Within 1 year of implementation, the transition programme improved some aspects of patients’ experiences with care delivery, such as the provision of opportunities for adolescents to visit the clinic alone (without parents; p < 0.001) and to decide who should be present during consultations (p < 0.05; Table 2). Other aspects did not improve within a 1-year period.

**Table 2:** Adolescents’ perceptions of current care (n = 242)

	**Mean (SD) at T0**	**Mean (SD) at T1**	**Change (T0 – T1) (SD)**	***p***
Has staff who know how to talk and listen to teenagers	5.7 (1.7)	5.7 (1.5)	0.0 (1.9)	0.813
Treats me as an individual and understands my specific needs	5.5 (1.7)	5.4 (1.8)	0.1 (2.2)	0.312
Has staff who understand the realities of being a teenager	5.6 (1.7)	5.4 (1.7)	0.1 (2.1)	0.364
Providers work well together	5.5 (1.6)	5.3 (1.8)	0.2 (2.0)	0.092
Is interested in me as a person, not just the illness	5.3 (1.8)	5.2 (1.8)	0.1 (2.3)	0.454
Allows me to make my own decisions about health care options in my own time	5.3 (1.8)	5.2 (1.9)	0.1 (2.3)	0.337
Gives me opportunities to be seen in the clinic alone (if i want to)	5.4 (2.0)	5.8 (1.7)	-0.4 (2.0)	<0.001
Provides information to other professionals involved in my health care (e.g. family doctor)	4.8 (1.8)	4.7 (1.8)	0.1 (2.3)	0.673
Allows me to decide who should be in the consultation/examination room	4.6 (2.2)	5.0 (2.0)	-0.4 (2.5)	<0.05
Helps me to prepare for my move to adult services	4.4 (1.9)	4.5 (2.0)	-0.1 (2.4)	0.470
Helps me to plan for my future	4.1 (1.9)	4.2 (2.0)	0.0 (2.4)	0.913
Providers from paediatric and adult care arrange joint appointments/consulting hours for adolescents	4.1 (2.0)	4.0 (2.1)	0.1 (2.5)	0.423
Helps me to improve independence by using an action plan	4.0 (2.0)	4.0 (1.9)	0.0 (2.4)	0.848
Does not waste my time at the clinic	4.3 (2.0)	4.2 (1.9)	0.1 (2.4)	0.626
Has staff who i can talk to about sensitive or difficult issues	4.9 (2.0)	5.0 (1.9)	-0.1 (2.3)	0.575
Has a specific staff member who is coordinating my transitional care	3.7 (1.9)	4.0 (1.9)	-0.3 (2.3)	0.083

SD, standard deviation; T0, beginning of ‘On Your Own Feet Ahead!’ quality improvement programme; T1, one year later. Only data from respondents who completed the questionnaire at both T0 and T1 were included in the analyses.

## Discussion

This study evaluated the effectiveness of transition programme implementation in reducing bottlenecks in transitional care (perceived by professionals) and improving the quality of (and chronically ill adolescents’ experiences with) care delivery within a 1-year period. The ability of transition programmes to improve care delivery experiences remains unproven; While and colleagues [2] concluded that ‘overall, the strength of the evidence was fairly weak’. More recent reviews also concluded that studies on the subject remain sparse [6,7] and more research that investigates the experiences of adolescents with the transition from paediatric to adult care is needed [29]. Thus, despite numerous compelling arguments for the implementation of transition programmes for chronically ill adolescents, many practitioners, researchers, and policymakers are uncertain of their effectiveness in eliminating bottlenecks in care delivery, and thereby improving the quality of care. Furthermore, the implementation of such programmes has not been described for the full range of adolescents with various chronic conditions.

Results from this study clearly showed that the transition programmes effectively reduced all bottlenecks identified by professionals at the start of the programme within a 1-year period. The most pronounced improvements were made in the coordination of care, access to resources for joint care services, and existence of a joint mission. Participating teams worked on synchronising paediatric and adult treatment protocols and procedures and organising joint clinics, and improving the provision of medical and psychosocial information about the transition period to patients.

The ‘On Your Own Feet Ahead!’ programme followed the Breakthrough Series quality improvement and implementation strategy [20]. The success of transition programmes is known to depend on the ability to effectively align interventions to the specific needs of chronically ill adolescents [1]. Use of a ‘bottom up approach’ within such programmes seems to be a key aspect because it augments participating professionals’ autonomy and ownership with respect to the selection, testing, and adjustment of interventions to local settings [30]. Strong features of this strategy are the efficient use of participating experts and the exchange of best practices for improvement [31]. Implementation involves professionals in Plan-Do-Study-Act (Deming) cycles and small-scale testing, and is based on perceived bottlenecks in care delivery. Professionals in this study may have reduced such bottlenecks by following this strategy, for example by learning from each other during the seven learning sessions. This finding underscores the importance of using a ‘bottom up approach’ for the identification and reduction of bottlenecks to improve transitional care.

Although professionals identified improvements in all areas, patients reported that transition programmes effectively improved some aspects of the organisation of care within a 1-year period. Improvements were made in the provision of opportunities for adolescents to visit the clinic alone and to decide who should be present during consultations, which were closely linked to the core interventions implemented in most hospital teams (e.g. encouraging independent consultations). These findings align with professionals’ reports that parents became more willing to cede control to adolescents, and that adolescents took more responsibility for self-care, over the 1-year period. Since not all implemented interventions are directly noticeable for patients, changes in the system of care delivery are known to be experienced first by professionals, followed by patients [32]. Hence, the quality of care and changes therein will predict more positive experiences of chronically ill patients over time. While, patients did not perceive significant improvements in all areas of transitional care, we therefore expect that they will experience such improvements over a longer period of time (e.g. 2 years). This study revealed some positive short term effects, and we expect that the programme will have more beneficial effects in the long term.

While this study did not find evidence on how improvements were achieved, other research clearly showed that gains in quality of care delivery were primarily improved by raising the quality of communication and task integration among professionals from diverse disciplines who share common objectives. These findings have implications for quality improvement efforts by collaborative care teams such as in transitional care where professionals from various occupational backgrounds have to work together to improve quality of care delivery. Diverse health care professionals must be strongly connected to provide effective, holistic care [33], which may also have contributed to improvement in transitional care in this study.

### Strengths and limitations

The strengths of this study include the longitudinal evaluation of a programme to improve transitional care for adolescents with a variety of conditions. Our research also provides more insight into the interventions used in transitional care practice and information about providers’ and young patients’ experiences with care. However, some limitations of this study should be taken into account. First, our findings provide no insight into which [set(s) of] intervention(s) had the greatest impact on (which) experiences with care. Moreover, the interventions that we studied were implemented within the usual consultation approach. We did not evaluate additional possibilities for counselling such as specific psychosocial treatments for children and youth [34].

Second, the low response rate among adolescents may have introduced non-response bias. Although the Mind the Gap instrument we used to investigate experiences with transitional care is well validated among chronically ill adolescents [28], the low response rate may have led to non-response bias. In some cases use of proxy information from caregivers is also useful, but important differences between adolescents’ and caregivers’ views have been reported [21]. Furthermore, it would have been possible to conduct interviews or ask adolescents to actively participate in the evaluation study. Previous research, however, showed that involving adolescents in participatory research to evaluate health care services is difficult [35].

## Conclusion

The implementation of transition programmes in the Netherlands following the Breakthrough Series approach reduced bottlenecks in care delivery as perceived by professionals. Patients perceived minor improvements in experiences with care delivery. This study demonstrated that transitional care interventions may improve the organisation and coordination of transitional care within a 1-year period. By setting specific goals based on experiences with bottlenecks, the Breakthrough approach helped hospital-based teams to improve transitional care delivery for adolescents with chronic conditions.

## Competing interests

The authors declare that they have no competing interests and confirm all patient/personal identifiers have been removed or disguised so the patient/person(s) described are not identifiable and cannot be identified through the details of the story.

## Authors’ contributions

AN, MR, AS and MS drafted the design for data gathering. AN, MR, AS, HS and MS were involved in acquisition of subjects and data. MS, HS, JC and AN performed statistical analysis and interpretation of data. AN drafted the manuscript and JC, MR, AS and MS helped drafting the manuscript and contributed to refinement. All authors have read and approved its final version.

## Pre-publication history

The pre-publication history for this paper can be accessed here:

http://www.biomedcentral.com/1472-6963/14/47/prepub

## Supplementary Material

Additional file 1 Overview of transitional care interventions selected by participating hospital teams in the ‘On Your Own Feet Ahead!’ quality improvement collaborative at T1 (n = 22). Click here for file
