# Supplementary material for: Reducing bottlenecks: professionals’ and adolescents’ experiences with transitional care delivery
Source: BMC Health Serv Res. 2014 Jan 31;14:47. doi: 10.1186/1472-6963-14-47 (PMC3913627; doi:10.1186/1472-6963-14-47)
Supplement: Additional file 1 — Overview of transitional care interventions selected by participating hospital teams in the ‘On Your Own Feet Ahead!’ quality improvement collaborative at T1 (n = 22). [file 1472-6963-14-47-S1.docx]

| **Additional file 1**  Overview of transitional care interventions selected by participating hospital teams in the ‘On Your Own Feet Ahead!’ quality improvement collaborative at T1 (n=22) | | | | | | | | | | | | |
| --- | --- | --- | --- | --- | --- | --- | --- | --- | --- | --- | --- | --- |
|  | ***Interventions to enhance self-management*** | | | | | | ***Interventions to improve organisation of transitional care*** | | | | | |
| **Teams** | **Informational brochure/website for youth** | | **Individual Transition Plan** | **Professional checklist for transition / RTP** | **Patient Reported Outcomes (Quality of Life)** | **Independent consultations** | **Transition protocol** | **Transition coordinator** | **Joint policy & mission PC - AC** | **Joint transition clinic / youth clinic** | **Structural consultation between PC - AC** | **Group consultations / group education** |
| **2009 – Testing Phase** |  | |  |  |  |  |  |  |  |  |  |  |
| Erasmus MC - Rotterdam  *Rheumatology* |  | | X | X | X | X | X | X | X | X | X |  |
| Erasmus MC – Rotterdam  *Neuromuscular disorders /Chronic ventilation* | X | | X | X |  | X | X | X | X | X | X |  |
| Radboud University Nijmegen Medical Centre – Nijmegen *Cystic Fibrosis* | X | |  | X | X |  | X | X | X | X | X |  |
| Catharina Hospital – Eindhoven  *Diabetes* |  | |  | X | X |  | X | X |  | X | X | X |
| Amphia Hospital – Breda  *Diabetes* |  | |  | X |  | X | X | X |  | X | X | X |
| Meander MC – Amersfoort  *Diabetes* |  | |  | X |  | X | X | X |  |  | X |  |
| Academic Medical Centre – Amsterdam *HIV* |  | |  | X |  | X | X |  | X | X | X |  |
|  |  | | | | | |  | | | | | |
|  |  | | | | | |  | | | | | |
|  |  | | | | | |  | | | | | |
|  | ***Interventions to enhance self-management*** | | | | | | ***Interventions to improve organisation of transitional care*** | | | | | |
| **Team**  *Condition* | **Information leaflet / website for youth** | **Individual Transition Plan** | | **Professional checklist for transition / RTP** | **Patient-reported outcomes (quality of life)** | **Independent consultations** | **Transition protocol** | **Transition coordinator** | **Joint policy & mission PC - AC** | **Joint transition clinic/youth clinic** | **Structured consultation between PC - AC** | **Group consultations/**  **education** |
| **2010 – Round 1** |  |  | |  |  |  |  |  |  |  |  |  |
| Erasmus MC – Rotterdam  *Cystic fibrosis* |  | X | | X | X | X |  |  | X | X | X |  |
| Sint Maartenskliniek – Nijmegen *Rheumatology* | X | X | | X |  |  | X |  | X | X | X |  |
| Radboud University Nijmegen Medical Centre – Nijmegen *Nephrology* | X | X | | X |  | X | X | X | X | X | X | X |
| Radboud University Nijmegen Medical Centre – Nijmegen *Urology* | X | X | | X |  |  | X |  |  |  |  |  |
| VUmc University Medical Center – Amsterdam  *Diabetes* |  | X | | X | X | X |  |  |  | X | X | X |
| Maasstad Hospital – Rotterdam *Diabetes* | X | X | | X |  | X |  |  |  | X | X | X |

|  | ***Interventions to enhance self-management*** | | | | | ***Interventions to improve organisation of transitional care*** | | | | | |
| --- | --- | --- | --- | --- | --- | --- | --- | --- | --- | --- | --- |
| **Teams** | **Information leaflet / website for youth** | **Individual Transition Plan** | **Professional checklist for transition / RTP** | **Patient Reported Outcomes (Quality of Life)** | **Independent consultations** | **Transition protocol** | **Transition coordinator** | **Joint policy & mission PC - AC** | **Joint transition clinic / youth clinic** | **Structural consultation between PC - AC** | **Group consultations / group education** |
| **2011 – Round 2** |  |  |  |  |  |  |  |  |  |  |  |
| Antonius Hospital – Sneek  *Diabetes* | X | X | X | X | X |  |  | X | X | X | X |
| Scheper Hospital – Emmen  *Diabetes* | X | X | X | X | X |  |  |  | X | X | X |
| Isala Clinics – Zwolle  *Diabetes* | X | X | X |  | X | X |  |  | X | X | X |
| Jeroen Bosch Hospital – ‘s Hertogenbosch *Diabetes* | X | X | X | X |  |  |  | X | X | X | X |
| Atrium MC – Heerlen  *Diabetes* |  | X | X | X | X |  | X |  | X | X |  |
| Maastricht UMC+ - Maastricht  *Diabetes* |  | X | X | X | X |  | X |  | X | X |  |
| Academic Medical Centre – Amsterdam *Nephrology* | X | X |  | X |  | X |  |  |  | X |  |
| VUmc University Medical Center – Amsterdam *Nephrology* | X | X |  | X | X | X |  |  | X | X | X |
| UMC Utrecht – Utrecht  *Nephrology* |  | X |  |  |  | X | X |  |  | X | X |

**Short descriptions of interventions**

***Interventions to enhance self-management***

Information leaflet / website for youth Written information for young people about transition of care

Individual Transition Plan Individual self-management plan for goal setting in several domains: filled out by adolescents (and parents)

Professional checklist for transition Checklist for professionals to record adolescents’ development in different domains

Patient Reported Outcomes (Quality of Life) Administration of instruments measuring patient-reported quality of life during consultations

Independent consultations Young people > 16 years of age attend consultations independently (without parents)

***Interventions to improve organisation of transition***

Transition protocol Written plan and schedule of professionals’ tasks and responsibilities during transition and transfer

Transition coordinator Dedicated paediatric or adult care professional responsible for overseeing adolescents’ transitions

Joint policy & mission PC - AC Definition of a joint mission between paediatric and adult care and alignment of procedures and treatment protocols

Transition clinic / youth clinic Joint clinic where paediatric and adult care professionals see patients together; dedicated clinic for young adults

Structural consultation between PC - AC Regular multidisciplinary discussions among paediatric and adult care professionals about transitioning patients

Group consultations / group education Group patient sessions (shared medical appointments or peer education sessions)
